# Clinical Efficacy and Adverse Reactions of Bevacizumab plus Radiochemotherapy in the Treatment of Advanced Gastric Cancer

**DOI:** 10.1155/2022/4900037

**Published:** 2022-02-08

**Authors:** Fang Wei

**Affiliations:** Digestive Department, Cangzhou Central Hospital, Cangzhou, China

## Abstract

**Objective:**

To evaluate the clinical efficacy and adverse reactions of bevacizumab plus radiochemotherapy in the treatment of advanced gastric cancer.

**Methods:**

Eighty-six eligible patients with gastric cancer treated in our institution from August 2019 to August 2020 were recruited and concurrently randomly assigned via the random number table method at a 1 : 1 ratio to receive neoadjuvant radiochemotherapy (control group) or bevacizumab (given on the first day of each course of neoadjuvant radiochemotherapy) plus neoadjuvant radiochemotherapy (study group) for 9 weeks (3 weeks as one course). Outcomes include the clinical efficacy and serum tumor marker levels before and after treatment. Patients in both groups were followed up for 12 months after treatment to obtain progression-free survival (PFS). Assessment of patients' quality of survival was done by the Karnofsky performance score (KPS). The occurrence of adverse reactions in patients during treatment was monitored to evaluate the safety of the treatment protocol.

**Results:**

The research group outperformed the control group significantly in terms of total treatment efficiency (*P* < 0.05). After treatment, serum carcinoembryonic antigen (CEA) and glycoantigen 199 (CA199) levels were markedly reduced in both groups, with lower results observed in the research group (*P* < 0.05). The research group had a significantly longer median PFS than the control group (95% CI: 1.182–2.367, 1.132–2.469, *P* < 0.05). A significantly higher improvement in quality of survival was observed in the research group than in the control group (*P* < 0.05). No significant intergroup differences in adverse reactions were reported, and no new safety signals were identified (*P* > 0.05).

**Conclusion:**

Bevacizumab potentiates the treatment outcomes for advanced gastric cancer by effectively attenuating the abnormalities of serum tumor marker levels and prolonging survival, with a high safety profile, in combination with radiochemotherapy versus radiochemotherapy alone.

## 1. Introduction

China has a high incidence of gastric cancer, with annual new cases accounting for more than 42% of the total cases in the world [1]. Symptoms of gastric cancer are nonspecific and insidious in the early stage, and disease progression will result in symptoms such as epigastric distention and pain, weight loss, nausea, and vomiting [2]. It has been reported [3] that about 60–80% of gastric cancer cases are in the advanced stages at the time of diagnosis, where patients are usually inoperable. Neoadjuvant radiochemotherapy is a preoperative treatment regimen for patients with locally advanced malignancies that contributes to inhibiting metastases and enhancing surgical effectiveness. Relevant clinical research has shown an excellent clinical benefit of neoadjuvant radiochemotherapy for patients with locally progressive gastric cancer [4]. The oxaliplatin-based FOLFOX4 chemotherapy regimen at 2-week intervals is highly effective and well-tolerated, which has been extensively used in clinical practice in recent years [5]. Molecularly targeted drugs are associated with better quality of survival of patients with malignancies. Bevacizumab is a humanized immunoglobulin G1 (IgG1) monoclonal antibody to the recombinant humanized vascular endothelial growth factor (VEGF). Several studies have demonstrated the efficacy and safety of bevacizumab combined with radiochemotherapy in locally advanced malignancies [6, 7]. However, the clinical efficacy and safety of bevacizumab plus radiochemotherapy for the treatment of gastric cancer are marginally explored. Accordingly, 86 gastric cancer patients assessed for eligibility treated in our institution from August 2019 to August 2020 were recruited for analysis. The results are as follows.

## 2. Materials and Methods

### 2.1. Clinical Data

Eighty-six eligible gastric cancer patients admitted to our hospital from August 2019 to August 2020 were identified as study subjects. Inclusion criteria were as follows: patients aged 18–80 years; patients who were diagnosed with gastric cancer diagnostic criteria by imaging and biopsy pathology [7], in TNM stages IIIb to IV; patients with measurable lesions by computed tomography (CT) or magnetic resonance imaging (MRI); patients with an expected survival time of ≥3 months; patients with Karnofsky performance status (KPS) score of ≥60 points; and patients without a history of antitumor treatment. Exclusion criteria were as follows: patients with abnormal coagulation function or important organ insufficiencies such as the heart, liver, and kidney; patients with cognitive impairment or mental impairment that prevented their cooperation in completing the investigation; patients with allergies or a history of previous drug allergy or allergy to the drugs used in this study; patients in lactation or pregnancy; and patients who had undergone palliative radiotherapy or chemotherapy before randomization. Eligible patients were concurrently randomly assigned via the random number table method to either the research group (*n* = 43) or the control group (*n* = 43). This study was approved by the hospital ethics committee, and the patients all signed the informed consent form.

### 2.2. Methods

#### 2.2.1. Control Group

Neoadjuvant radiochemotherapy [8, 9] was performed, with 1 course of treatment every 3 weeks for 3 courses. Radiotherapy: extracorporeal irradiation was carried out using high-energy X-rays, with irradiation fields of 2-3 cm outside the primary lesion, intraretinal lymph nodes in the greater gastric curvature, intraretinal lymph nodes in the lesser curvature, and pyloric lymph nodes, 180 cGy/time, 1 time/day, 5 times/week, total radiation 3000 cGy–4000 cGy/3-4 weeks. Chemotherapy: chemotherapy, using the FOLFOX4 regimen, was administered concurrently with radiotherapy. On the first day of each course, oxaliplatin (Nanjing Pharmaceutical Factory Co., Ltd., State Drug Quantifier H20000686), 85 mg/m^2^, was administered intravenously within 2 h. For the first 2 days of each course, calcium folinic acid for injection (Shanxi Pude Pharmaceutical Co., Ltd., State Pharmacopoeia H14022465), 200 mg/m^2^, was administered by intravenous drip for 2 h, followed by administration of fluorouracil injection (Liaoning Xingao Pharmaceutical Co., Ltd., State Pharmacopoeia H21024236), 400 mg/m^2^, by intravenous push and 600 mg/m^2^ by intravenous pump for 22 h.

#### 2.2.2. Research Group

On the basis of the control group, bevacizumab (Qilu Pharmaceutical Co., Ltd., State Drug Quantifier S20190040), 7.5 mg/kg, intravenous drip, was given on the first day of each course, 1 time per course for 3 courses of treatment.

### 2.3. Observation Indexes

#### 2.3.1. Clinical Efficacy [10]

Complete response (CR): the tumor disappears after treatment and lasts for more than 4 weeks. Partial response (PR): the total reduction of the longest diameter of the tumor lesion is more than 50% after treatment and lasts for more than 4 weeks. Stable disease (SD): the total reduction of the longest diameter of the tumor lesion is 25–50% after treatment and lasts for more than 4 weeks. Progressive disease (PD): the sum of the longest diameter of tumor lesions increases by more than 20% after treatment or new lesions appear. Total efficacy = CR + PR + SD.

#### 2.3.2. Serum Tumor Markers

Before and after treatment, 5 mL of fasting elbow venous blood was collected from patients and centrifuged at 3000 r/min for 10 min to separate the serum. Serum carcinoembryonic antigen (CEA) and carbohydrate antigen 199 (CA199) levels were determined by ELISA, and the kits were purchased from Guangzhou Bohui Biotechnology Co. (item no. FT-P33703R), with uniform lot numbers and batches.

#### 2.3.3. Survival

Patients in both groups were followed up for 12 months after treatment by outpatient follow-up or telephone follow-up, and the progression-free survival (PFS), the time from randomization to disease progression or death from any cause, was recorded.

#### 2.3.4. Improvement in Quality of Survival

An increase in KPS score of 10 points or more after treatment was considered an improved life quality, an increase in KPS score or decrease within 10 points after treatment was considered stable life quality, and a decrease in KPS score of more than 10 points after treatment was considered decreased life quality.

#### 2.3.5. Safety Evaluation

Patients were monitored for the occurrence of adverse reactions during treatment, including nausea and vomiting, thrombocytopenia, leukopenia, peripheral neurotoxicity, abnormal liver and kidney function, and bone marrow suppression. The adverse reactions in this study were stratified into grades I to IV. Grade I as per the National Cancer Institute Common Terminology Criteria for Adverse Events v3.0 (NCI-CTCAEv3.0) [11]: adverse reactions are tolerated by patients, do not require discontinuation or dose reduction, can be relieved without treatment or with general symptomatic treatment, and have no direct impact on the patients' recovery. Grade II: adverse reactions are intolerable to the patient, require discontinuation of medication or dose reduction, can be alleviated by general symptomatic treatment, and have no direct effect on the patients' recovery. Grade III: patients with obvious symptoms of adverse reactions, abnormal relevant examination, or combined with pathophysiological changes in other organs, require discontinuation of medication, which directly impacts the patient's recovery, or the adverse reaction lasts for more than 7 days. Grade IV: adverse reactions threaten the patient's life, require immediate emergency treatment and discontinuation of medication, or adverse reactions persist for more than 30 days.

### 2.4. Statistical Analyses

SPSS 20.0 software was used for the statistical analyses of the data. The measurement data conforming to normal distribution were expressed as ($\bar{x}\pm s$), and the *t*-test for independent samples was used for comparison between two groups, and the *t*-test for paired samples was used for intragroup comparison. Count data were expressed as frequencies or composition ratios, with total cases ≥40 and minimum theoretical frequency >5, using the chi-square uncorrected method. Kaplan–Meier curve was used to analyze the survival status of patients. Differences were considered statistically significant at *P* < 0.05.

## 3. Results

### 3.1. Comparison of General Data

In the research group, there were 23 males and 20 females, aged 38–75 years, with a mean age of (54.38 ± 3.52) years, KPS score of (68.94 ± 2.13) points, and disease duration of (5.47 ± 2.32) years. The research group had 26 cases of TNM stage IIIb and 17 cases of stage IV, 7 cases of mucinous carcinoma, 3 cases of indolent cell carcinoma, and 33 cases of adenocarcinoma in terms of pathological type and 8 cases of undifferentiated, 13 cases of lowly differentiated, 12 cases of moderately differentiated, and 10 cases of highly differentiated in terms of differentiation. In the control group, there were 25 males and 18 females, aged 39–78 years, with a mean age of (55.12 ± 3.57) years, KPS score of (69.13 ± 2.15) points, and disease duration of (5.54 ± 2.34) years. The control group had 28 cases of TNM stage IIIb and 15 cases of stage IV, 8 cases of mucinous carcinoma, 4 cases of indolent cell carcinoma, and 31 cases of adenocarcinoma in terms of pathological type and 7 cases of undifferentiated, 11 cases of lowly differentiated, 13 cases of moderately differentiated, and 12 cases of highly differentiated in terms of differentiation. There was no statistically significant difference in the general data between the two groups of patients (*P* > 0.05), as given in Table 1.

### 3.2. Comparison of Clinical Efficacy

The total efficacy of treatment in the research group was significantly higher than that in the control group (*P* < 0.05), as given in Table 2.

### 3.3. Comparison of Serum Tumor Marker Levels

There was no statistically significant difference between the serum CEA and CA199 levels of patients in the two groups before treatment (*P* > 0.05). After treatment, serum CEA and CA199 levels were markedly reduced in both groups, with lower results observed in the research group (*P* < 0.05), as given in Table 3.

### 3.4. Comparison of PFS

**Figure 1:**
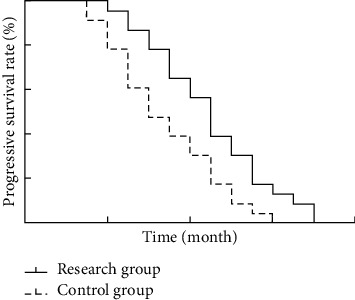
Comparison of survival between two groups of patients.

All study subjects received complete follow-up with complete and uncompromised case data. The median PFS was 8.34 months and 5.48 months in the research group and the control group, respectively. The median PFS was significantly longer in the research group than in the control group (95% CI: 1.182–2.367, 1.132–2.469, *P* < 0.05), as shown in Figure 1.

### 3.5. Comparison of Survival Quality

No statistically significant differences were found in the stabilization and reduction rates of survival quality between the two groups (*P* > 0.05). The improvement rate of survival quality in the study group was significantly higher than that in the control group (*P* < 0.05), as given in Table 4.

### 3.6. Safety Evaluation

No reported grade IV adverse events were found. The differences were not statistically significant in the total incidence of nausea and vomiting, thrombocytopenia, leukopenia, peripheral neurotoxicity, abnormal liver and kidney function, and bone marrow suppression between the two groups (*P* > 0.05), as given in Table 5.

## 4. Discussion

Gastric cancer is a common clinical malignancy with high morbidity and mortality [12]. The early symptoms of gastric cancer are insidious and nonspecific, and the disease has usually progressed to the advanced stages at the time of diagnosis, which results in poor prognosis [13]. Research has shown that the postoperative five-year survival rate is approximately 90% for patients with early gastric cancer and down to less than 30% for patients in the advanced stage [14]. According to the latest NCCN and CSCO oncology treatment guidelines, neoadjuvant therapy prior to radical surgery is considered a key component of the standard treatment regimen for gastric cancer. It has been reported that neoadjuvant therapy contributes to achieving tumor downstaging and negates the inoperability of patients [15]. Subgroup analysis suggested the benefits of adjuvant radiochemotherapy in patients with lymph node-positive and intestinal gastric cancer versus adjuvant chemotherapy alone [16].

In recent years, with the in-depth research on antibodies, immunoglobulins, and hybridoma technology, some monoclonal antibody drugs with targeted effects against gastric cancer have received extensive attention [17]. Bevacizumab is a representative IgG1-type monoclonal antibody in targeted therapy for gastric cancer. One study found that bevacizumab combined with chemotherapy could effectively suppress the expression level of tumor markers in patients with advanced gastric cancer and improve the overall efficiency of treatment with safety benefits [18]. Moreover, the joint treatment of bevacizumab and radiotherapy has been reported to improve the short-term efficacy and prolong PFS and overall survival in patients with glioma without increasing the risk of toxic side effects [19]. Accordingly, this study applied bevacizumab in neoadjuvant radiochemotherapy for advanced gastric cancer, and the results showed that the CR rate, PR rate, and SD rate in the study group were 6.98%, 55.81%, and 6.98%, respectively, with a total efficacy of 69.77%, which were superior to the results reported by Yang and Dong (65.22%) [19], and the CR rate, PR rate, and SD rate in the control group were 2.33%, 20.93%, and 16.28%, respectively, with a total efficacy of 39.53%, which was lower than the results reported by Li et al. [20]. The superiority of results versus those of previous studies may be attributed to the difference in the radiochemotherapy regimens, which will be further explored in future studies. The higher overall efficiency and median PFS provided by the addition of bevacizumab suggested that bevacizumab plus radiochemotherapy for advanced gastric cancer could significantly improve the efficacy and prolong the survival of patients, with more clinical benefits versus radiochemotherapy alone. Analysis of serum tumor marker levels revealed that both treatment regimens were effective in reducing serum CEA and CA199 levels in patients with advanced gastric cancer, in which the joint treatment yielded more features favoring patients' prognosis, as evidenced by the remarkably higher improvement of survival quality at follow-up in the study group than that in the control group. The safety evaluation found no reported grade IV adverse events after treatment of bevacizumab combined with radiochemotherapy. There was no statistically significant difference in the overall incidence of nausea and vomiting, thrombocytopenia, leukopenia, peripheral neurotoxicity, abnormal liver and kidney function, and bone marrow suppression between the two groups, and no new safety signals were identified, indicating a higher safety profile of the joint treatment. Bevacizumab plus radiochemotherapy can be prioritized according to the patient's clinical situation.

In conclusion, bevacizumab potentiates the treatment outcomes for advanced gastric cancer by effectively attenuating the abnormalities of serum tumor marker levels and prolonging survival, with a high safety profile, in combination with radiochemotherapy versus radiochemotherapy alone. This study was limited by a short follow-up period, so whether patients with advanced gastric cancer can benefit from the combined regimen in the long run requires further investigation with extended follow-up periods.

## Figures and Tables

**Table 1:** Comparison of general data between the two groups of patients.

Indexes	Research group (*n* = 43)	Control group (*n* = 43)	*t*/*χ*^2^	*P*
Age (year, $\bar{x}\pm s$)	54.38 ± 3.52	55.12 ± 3.57	0.968	0.336
KPS scores (points, $\bar{x}\pm s$)	68.94 ± 2.13	69.13 ± 2.15	0.412	0.682
Course of disease (year, $\bar{x}\pm s$)	5.47 ± 2.32	5.54 ± 2.34	0.139	0.890
Gender (*n*/%)			0.189	0.664
Male	23	25		
Female	20	18		
TNM stage (*n*/%)			0.199	0.655
IIIb	26	28		
IV	17	15		
Pathological type (*n*/%)			0.272	0.873
Mucinous carcinoma	7	8		
Indolent cell carcinoma	3	4		
Adenocarcinoma	33	31		
Differentiation (*n*/%)			0.455	0.929
Undifferentiated	8	7		
Lowly differentiated	13	11		
Moderately differentiated	12	13		
Highly differentiated	10	12		

KPS, Karnofsky performance score (KPS); TNM, tumor node metastasis.

**Table 2:** Comparison of clinical efficacy between the two groups (*n*/%).

Groups	*n*	CR	PR	SD	PD	Total efficacy
Research group	43	3/6.98	24/55.81	3/6.98	13/30.23	30/69.77
Control group	43	1/2.33	9/20.93	7/16.28	26/60.47	17/39.53
*χ* ^2^						7.929
*P*						0.005

CR, complete remission; PR, partial remission; SD, stable disease; PD, progressive disease.

**Table 3:** Comparison of serum tumor marker levels before and after treatment in the two groups ($\bar{x}\pm s$).

Groups	*n*	CEA (mg/L)		CA199 (mg/L)	
		Before treatment	After treatment	Before treatment	After treatment
Research group	43	66.59 ± 10.32	18.25 ± 4.14^*∗*^	413.78 ± 47.89	168.49 ± 28.35^*∗*^
Control group	43	67.14 ± 10.47	32.68 ± 3.89^*∗*^	415.35 ± 48.62	257.67 ± 29.51^*∗*^
*t*		0.245	16.657	0.151	14.291
*P*		0.807	≤0.001	0.881	≤0.001

^*∗*^ *P* < 0.05 in comparison with the same group before treatment. CEA, carcinoembryonic antigen; CA199, glycoantigen 199.

**Table 4:** Comparison of improvement in quality of survival between the two groups (*n*/%).

Groups	*n*	Improved	Stable	Decreased
Research group	43	18/41.86	19/44.19	6/13.95
Control group	43	9/20.93	24/55.81	10/23.26
*χ* ^2^		4.373	1.163	1.229
*P*		0.037	0.281	0.268

**Table 5:** Comparison of the incidence of adverse reactions in the two groups (*n*/%).

Adverse reactions	Research group (*n* = 43)				Control group (*n* = 43)			
	I	II	III	Total incidence	I	II	III	Total incidence
Nausea and vomiting	12	5	1	18/41.86	10	6	0	16/37.21
Thrombocytopenia	2	3	1	6/13.95	3	1	1	5/11.63
Leukopenia	7	10	4	21/48.84	8	8	3	19/44.19
Peripheral neurotoxicity	2	1	0	3/6.98	1	0	0	1/2.33
Abnormal liver and kidney function	9	6	2	17/39.53	7	7	0	14/32.56
Bone marrow suppression	7	5	1	13/30.23	8	3	0	11/25.58

## Data Availability

The datasets used to support the findings of this study are available from the corresponding author upon request.
